# Expression of Interleukin 17A and 17B in Gingival Tissue in Patients with Periodontitis

**DOI:** 10.3390/jcm12144614

**Published:** 2023-07-11

**Authors:** Małgorzata Mazurek-Mochol, Karol Serwin, Tobias Bonsmann, Małgorzata Kozak, Katarzyna Piotrowska, Michał Czerewaty, Krzysztof Safranow, Andrzej Pawlik

**Affiliations:** 1 Department of Periodontology, Pomeranian Medical University, 70-111 Szczecin, Poland; malgorzata.mazurek@poczta.onet.pl (M.M.-M.); tobias.bonsmann1@gmail.com (T.B.); 2 Department of Physiology, Pomeranian Medical University, 70-111 Szczecin, Poland; karol.serwin@pum.edu.pl (K.S.); piot.kata@gmail.com (K.P.); michal.czerewaty@wp.pl (M.C.); 3 Department of Dental Prosthetics, Pomeranian Medical University, 70-111 Szczecin, Poland; gosia-ko@o2.pl; 4 Department of Biochemistry and Medical Chemistry, Pomeranian Medical University, 70-111 Szczecin, Poland; chrissaf@mp.pl

**Keywords:** IL-17, periodontal disease, inflammation

## Abstract

Periodontitis (PD) is a chronic inflammatory disease that is initiated by oral microorganisms. The pathogens induce the production of cytokines, such as interleukin (IL)-17, which enhances the inflammatory response and progression of the disease. The aim of this study was to examine the expression and localization in gingival tissue of IL-17A and IL-17B in patients with periodontitis. This study included 14 patients with periodontal disease and 14 healthy subjects without periodontal disease as a control group. There were no statistically significant differences in the expression of *IL-17A* mRNA between patients with periodontitis and control subjects. The expression of *IL-17B* mRNA was statistically significantly lower in patients with periodontitis in comparison with healthy subjects (*p* < 0.048). The expression of *IL-17A* correlated significantly with the approximal plaque index. The *IL-17B* expression in gingival tissue correlated with the clinical attachment level. This correlation reached borderline statistical significance (*p* = 0.06). In immunohistochemical analysis, we have shown the highest expression of IL-17 protein in inflamed connective tissue, epithelium, and granulation tissue from gingival biopsy specimens from patients with periodontitis. In biopsy specimens from healthy individuals, no IL-17 was found in the epithelium, while an expression of IL-17 was found in the connective tissue. The results of our study confirm the involvement of IL-17 in the pathogenesis of periodontitis. Our results suggest that an increase in IL-17 protein expression in the gingival tissue of patients with periodontitis occurs at the post-translational stage.

## 1. Introduction

Periodontitis (PD) is a chronic inflammatory state caused by bacterial infection in the periodontal tissues. Susceptibility to periodontitis may be caused by genetic factors as well as environmental factors, such as smoking and oral hygiene [1]. In periodontal tissue, bacterial infections induce the immune response leading to the production of inflammatory mediators, such as proinflammatory cytokines, and subsequently to tissue destruction [2,3]. The body’s immune response to eliminate the bacterial infection can lead to an abnormal immune response causing the destruction of periodontal tissues, including the alveolar process. The cellular immune response plays an important role in the inflammatory process in PD, and Th17 cells and the IL-17 they produce are an important component of this response. IL-17 is a multidirectional cytokine and exists as six isoforms IL-17A-IL-17F. IL-17 is primarily produced by T helper 17 (Th17) cells, which also secrete other cytokines involved in the immune response. Previous studies have shown that interleukin (IL)-17 is a cytokine involved in the pathogenesis of PD and other inflammatory diseases [4,5,6]. IL-17 plays an important role in immune processes on the surfaces of mucous membranes, providing an important element of protection against bacterial infections. In the inflammatory state, IL-17 can affect various types of cells including endothelial cells, fibroblasts, osteoblasts, and keratinocytes [7,8,9]. It can stimulate these cells to synthesize numerous pro-inflammatory cytokines that aggravate the inflammatory process [10,11,12]. IL-17 can also inhibit osteoblasts, leading to the reduction in alveolar bone [13].

Many studies to date have shown an important role for IL-17 in periodontitis [14]. Previous studies have indicated elevated levels of IL-17, particularly in saliva and gingival fluid, in patients with periodontitis. However, these findings vary depending on patient selection criteria, disease severity, or diagnostic criteria. Increased levels of IL-17 were found, especially in patients with an aggressive form of periodontitis. To date, there have been few studies evaluating IL-17 in the gingival tissue of patients with periodontitis. In addition, most of the studies did not evaluate individual IL-17 isoforms, but only total IL-17. The results of previous studies suggest that IL-17A and IL-17B isoforms may play the most important role in the development of periodontitis [7,8,9].

The aim of this study was to examine the expression and localization in gingival tissue of IL-17A and IL-17B in patients with periodontitis.

## 2. Materials and Methods

### 2.1. Patients

This study included 14 patients with periodontitis (5 male, 9 female; mean age 54.2 ± 12.5 years) diagnosed according to the 2017 classification system of periodontal diseases [15], and 14 healthy subjects (5 male, 9 female; mean age 52.8 ± 11.3 years) without periodontal disease who had small oral surgery as a control group.

In patients with periodontitis, the gingival tissue for examination was obtained during flap procedures, gingivaosteoplasty, as well as through a distal wedge or trapezoidal excision at the last molar.

In patients with healthy periodontium, the gingival tissue was obtained during aesthetic lengthening of clinical tooth crowns before prosthetic, conservative, or orthodontic treatment. The procedure was performed under local anesthesia, during which the removed gingival tissue, normally disposed of, was used in our study.

The patients with diagnosed periodontitis were defined if interdental CAL ≥ 2 mm was detectable at two or more than two non-adjacent teeth or buccal or oral CAL ≥ 3 mm and periodontal pockets > 3 mm were detectable at two or more than two teeth and the observed CAL could not be attributed to non-periodontal causes [16]. We also observed bleed on probing with deep probing pocket depth (PPD ≥ 5 mm). A minimum of 15% radiographic bone loss was required. When more than 30% of the teeth were affected, periodontitis was considered as generalized in relation to extent and distribution.

Exclusion criteria were as follows: (1) smoking within the past 5 years; (2) antibiotic therapies during the previous 6 months; (3) pregnancy; (4) chronic apical periodontitis; and (5) any systemic condition that could affect the progression of periodontitis (e.g., immunologic disorders, diabetes, and osteoporosis).

Gingival tissues were collected using a scalpel. The tissue samples were taken from a single tooth in each participant. The excised tissues were washed with PBS and immediately placed in a commercial reagent for RNA isolation for gene expression assays. The study was approved by the local ethics committee (BN-001/93/08) and is in accordance with the Declaration of Helsinki. Patients were informed about the study and their written consent was obtained.

### 2.2. Periodontal Examination

Periodontal evaluation included probing pocket depth (PPD), clinical attachment level (CAL), the approximal plaque index (API), and bleeding on probing (BoP).

Clinical measurements were taken in homogeneous conditions in a dental clinic. Probing pocket depth (PPD) and clinical attachment level (CAL) were assessed at six sites per tooth, using an UNC 15 periodontal probe calibrated with 1 mm (Hu-Friedy Mfg Co., Inc., Chicago, IL, USA). A UNC-15 Color-Coded Probe was used for all explorations. Pressure of approximately 20 g was applied for probing.

### 2.3. Quantitative Real-Time Reverse Transcription PCR (qRT-PCR) Analysis

Total RNA was extracted from 50–100 mg tissue samples using an RNeasy Lipid Tissue Mini Kit (Qiagen, Hilden, Germany) in accordance with the manufacturer’s protocol. The obtained RNA was used for the reverse transcription reaction. A quantity of 1 µg of RNA from each sample was reverse transcribed into cDNA with the RevertAid First Strand cDNA Synthesis Kit (Thermo Scientific, Waltham, MA, USA) according to the manufacturer’s instructions.

Quantitative assessment of mRNA levels was performed by real-time RT-PCR using an ABI 7500 Fast instrument with Power SYBR Green PCR Master Mix reagent. Real-time conditions were as follows: 95 °C (15 s), 40 cycles at 95 °C (15 s) and 60 °C (1 min).

In the next step, the 2^−ΔCt^ method was used to calculate the values. The values were normalized to the B2M (β2-microglobulin) gene. The primer sequences used in the study were prepared according to the sequence information obtained from the NCBI database, and were synthesized by Oligo.pl (IBB PAN, Warsaw, Poland). Primers used for gene expression analysis by qRT PCR were as follow: B2M-F 5′-AATGCGGCATCTTCAAACCT-3′, B2M-R 5′-TGACTTTGTCACAGCCCAAGA-3′, IL17A-F 5′-AGATTACTACAACCGATCCAC-3′, IL17A-R 5′-GGGGACAGAGTTCATGTGGT-3′, IL17B-F 5′-GAGCCCCAAAAGCAAGAGGA-3′, IL17B-R 5′-TGCGGGCATACGGTTTCATC-3′.

### 2.4. Immunohistochemical Analysis of IL17

After deparaffinization in Xylene (ChemPour, Tarnowskie Góry, Poland), sections of gingival or granulation tissue from the inflammatory site (3 μm thick) were hydrated in gradually decreasing ethanol (100–70%) and rinsed in tap water for 5 min. Next, heat epitope retrieval was performed in retrieval solution buffer pH = 6 (DAKO, Glostrup, Denmark), in a microwave oven for 10 min. After cooling to room temperature (RT), all the slides were incubated with 0.3% solution of H_2_O_2_ for inhibition of endogenous peroxidase, washed twice with PBS, and then incubated with 2.5% horse serum to prevent unspecific antibody bounding (Vector Laboratories, Newark, CA, USA). After incubation with serum, slides were incubated in a humid chamber with primary antibody of rabbit anti-human IL-17 (ab2) (Sigma-Aldrich, Burlington, MA, USA) for 1 h at RT. After washing in PBS, immunoreactions were visualized with ImmPRESS UNIVERSAL REAGENT and Vector NovaRED Substrate KIT FOR PEROXIDASE (Vector Laboratories, Newark, CA, USA) according to protocols provided by the manufacturer. Nuclei were counterstained with Mayer hematoxylin (Sigma-Aldrich, Burlington, MA, USA). As a negative control, the primary antibody was replaced with PBS on the specimen. Positive staining was defined by visual identification of a yellow/brown pigmentation in a bright field microscope. Images were collected with an Olympus IX81 inverted microscope (Olympus, Hamburg, Germany) with a color camera and with CellSens image processing software (Olympus, Germany).

### 2.5. Statistical Analysis

Non-parametric tests were used for statistical analysis of quantitative variables (*p* < 0.05; Shapiro–Wilk test). The Mann–Whitney U test was used for comparisons between two groups, and Spearman’s rank correlation coefficient for assessment of associations between variables. Normalized expression values are presented as medians with lower and upper quartiles (Q1–Q3). Values of *p* < 0.05 were considered to indicate statistical significance. The study had a statistical power of 80% to detect the true effect size corresponding to (1) the difference between group means equal to 1.3 standard deviations, and (2) the correlation coefficient between parameters measured in patients equal to ±0.67.

## 3. Results

**Figure 1:**
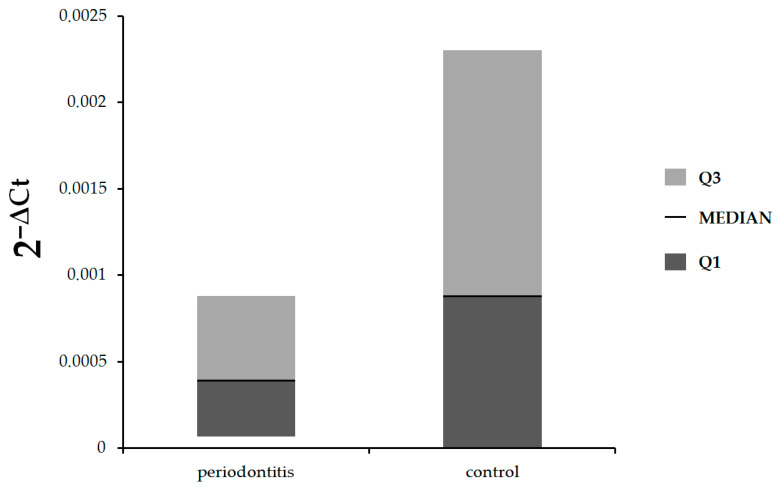
Expression of *IL-17A* mRNA in gingival tissue of patients with periodontitis and control subjects.

**Figure 2:**
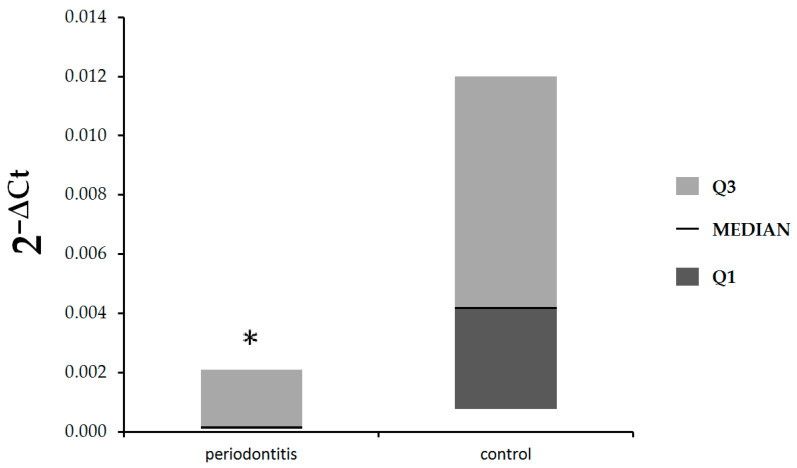
Expression of *IL-17B* mRNA in gingival tissue of patients with periodontitis and control subjects, * *p* = 0.048, Mann–Whitney U test.

In the first step of our study, we compared the expression of *IL-17A* and *IL-17B* at the mRNA level in gingival tissue in patients with periodontitis and control subjects. As shown in Figure 1, there were no statistically significant differences in expression of *IL-17A* between patients with periodontitis and control subjects (median: 0.00039, Q1–Q3: 0.000068–0.00088 vs. median: 0.00088, Q1–Q3: 0–0.0023, respectively, *p* = 0.81). The expression of *IL-17B* was statistically significantly lower in patients with periodontitis in comparison with healthy subjects (median: 0.00017, Q1–Q3: 0.000087–0.0021 vs. median: 0.0042, Q1–Q3: 0.00076–0.012, respectively, *p* = 0.048), (Figure 2).

Additionally, we examined the correlations between expression of *IL-17A* and *IL-17B* and clinical parameters such as PPD, API, CAL, and BoP. As shown in Table 1 the expression of *IL-17A* correlated significantly with API values. Moreover, the *IL-17B* expression in gingival tissue correlated with CAL values. This correlation reached borderline statistical significance (*p* = 0.06). There were no statistically significant correlations between the other clinical parameters noted above and expression of *IL-17A* and *IL-17B* in gingival tissues.

**Figure 3:**
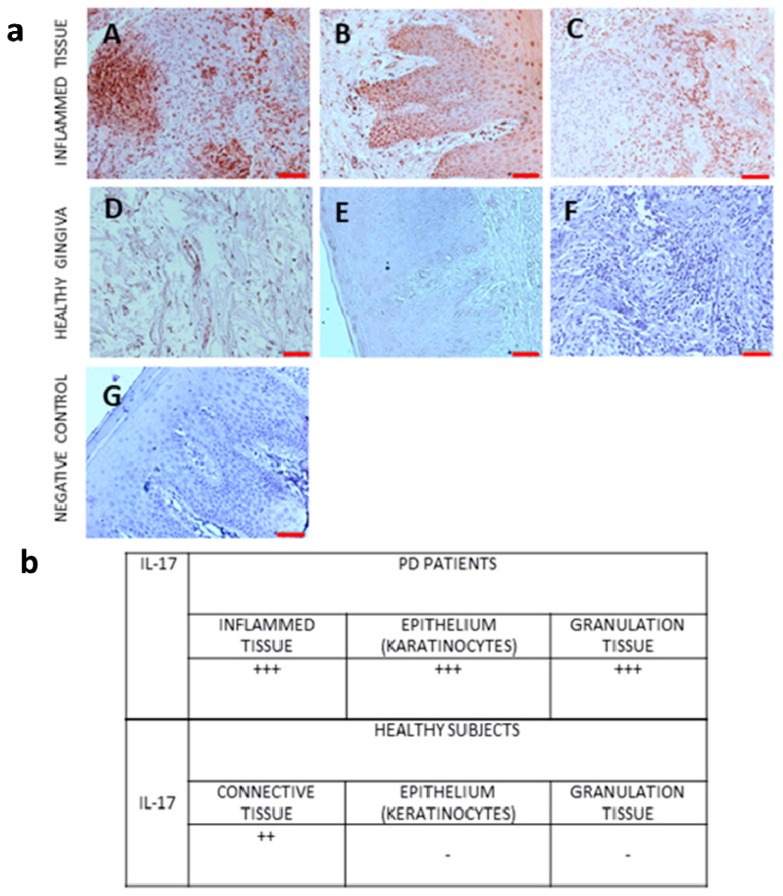
The expression of IL-17 protein in gingival tissue from: panel (**a**) (**A**–**C**): PD patients (inflamed tissue: (**A**)—connective tissue, (**B**)—epithelial tissue, (**C**)—granular tissue;); panels (**D**–**E**): controls (healthy gingiva): (**D**)—connective tissue, (**E**)—epithelial tissue, (**F**)—granular tissue; negative primary antibody control, where primary antibody was replaced with PBS (negative control—panel (**G**)). Original objective magnification ×20, scale bar = 50 µm, only representative images are presented. Panel (**b**): cellular immunolocalization of IL-17; +++ very high expression, ++ high expression, - no expression.

We also performed immunohistochemical analysis of IL-17 protein expression in gingival tissue from PD patients and controls. Figure 3 shows the expression of IL-17 protein in gingival tissue from PD patients and controls.

The expression of proinflammatory markers was observed in all samples of inflammatory tissues. The pigmentation was observed in the cytoplasm of cells in connective tissue and in keratinocytes. In the case of granulation tissue, the immunoreaction was observed in the cytoplasm of inflammatory cells. According to the saturation and color of pigmentation, the result was described as +++ very high expression, ++ high expression, - no expression.

## 4. Discussion

Periodontitis is a chronic inflammation of periodontal tissues initiated by a bacterial infection. However, mechanisms to eliminate the bacterial infection can develop into chronic inflammation that causes damage to periodontal tissues, including the alveolar process. Numerous immune response cells that secrete a number of pro-inflammatory mediators are involved in the development of this inflammation. Among the cells that play an important role in this process are Th-17 lymphocytes, which produce IL-17 [10,11]. IL-17 is an important component of the inflammatory cascade in periodontitis. It exhibits a number of direct actions in periodontal tissues, such as stimulating immune response cells to synthesize other cytokines (interleukin-6, interleukin-8, TNF and G-CSF) that are involved in the development of periodontitis [13].

IL-17 is an important cytokine that plays a multidirectional function in periodontal tissues. Under physiological conditions, IL-17 plays an important role in the mucosal barrier that protects against bacterial infections. IL-17 is mainly produced by Th-17 cells; however, the expression of this cytokine was also found on other immune cells such as neutrophils and natural killer cells [9,10]. IL-17 increases the recruitment and stimulation of other immune response cells, mainly neutrophils, which play an important role in the development of periodontitis. This cytokine also increases the synthesis of metalloproteinases, which cause periodontal tissue destruction. IL-17 influences the expression of RANKL and osteoprotegerin, thereby affecting the destruction of alveolar bone [10].

The role of IL-17 in the pathogenesis of PD has been investigated in animal models and in clinical studies [4,5,6]. Previous studies have indicated increased expression of IL-17 in serum, gingival cervical fluid, and saliva of patients with various forms of PD [4,5,6]; however, the studies examining the expression of IL-17 in gingival tissue are limited. These studies analyzed various forms of periodontal disease and periodontitis (aggressive and chronic according to the previous classification). Most of the studies evaluated total IL-17 without identifying the different isoforms of this cytokine. Most studies have shown increased expression of IL-17, especially in saliva and gingival fluid in patients with acute forms of periodontitis [17,18,19]. In the study by Awang et al., serum, saliva, and gingival crevicular fluid IL-17 levels were higher in periodontitis patients and correlated positively with clinical parameters of attachment loss, pocket depth, and bleeding on probing [20]. Liukkonen et al. indicated that salivary concentrations of IL-17 were elevated significantly in patients with local periodontal disease compared with controls and patients with generalized periodontal disease [21]. Furthermore, in the study by Mitani et al., IL-17 levels were significantly higher in gingival crevicular fluid from patients with periodontitis [6].

The studies also examined IL-17 serum concentrations in patients with PD [22,23]. Duarte et al. [14] reported significantly higher levels of IL-17 in the serum of patients with chronic PD. Similar results were presented by Cifcibasi et al., who also observed increased levels of IL-17 in patients with aggressive PD [24]. However, the mechanisms causing the increase in IL-17 in systemic circulation remain unclear. Duarte et al. demonstrated elevated serum IL-17 levels in patients with generalized aggressive periodontitis, which were reduced by therapy [25]. These studies suggest that local inflammatory changes in gingival tissue caused an increase in the IL-17 level in systemic circulation.

Increased numbers of IL-17-producing cells were detected in gingival tissue in patients with periodontitis [26]. These results suggest that the degree of infiltration with IL-17-producing cells is correlated with the severity of inflammation in PD.

In our study, we examined the expression of *IL-17A* and *IL-17B* at the mRNA level, as well as IL-17 protein expression in gingival tissue from patients with periodontitis as well as in healthy subjects. Our results did not detect statistically significant differences in *IL-17A* expression at the mRNA level in the gingival tissue of periodontitis patients and healthy subjects, but *IL-17A* expression correlated with the proximate plaque index. We have shown lower expression of *IL-17B* at the mRNA level in patients with periodontitis in comparison with healthy subjects. The gingival expression of *IL-17B* at the mRNA level correlated with clinical attachment loss. Clinical attachment loss is a parameter indicating the severity of periodontal disease. Clinical attachment loss is caused by inflammation and the secretion of cytokines, chemokines, metalloproteinases, and other mediators by cells of the immune system, causing the destruction of periodontal tissues. IL-17B has been shown to enhance neutrophil migration, thereby increasing inflammation [8]. It is possible that *IL-17B* expression at the mRNA level in gingival tissue may be inhibited by other mediators involved in the development of periodontitis.

In immunohistochemical analysis, we analyzed the IL-17 protein expression in gingival tissue from PD patients and healthy subjects. The highest expression of IL-17 was shown in inflamed connective tissue, epithelium (karatinocytes), and granulation tissue from gingival biopsy specimens from patients with periodontitis. In biopsy specimens from healthy individuals, no IL-17 was found in the epithelium (keratinocytes), while an expression of IL-17 was found in the connective tissue.

One limitation of our study is the lack of a positive control in immunohistochemical analysis.

The results of this study indicated that *IL-17A* expression in gingival tissue at the mRNA level did not differ between PD patients and controls, while *IL-17B* expression at the mRNA level is lower in PD patients than in controls. In contrast, we showed increased IL-17 expression at the protein level in the gingival tissue of PD patients compared to the healthy group. The results of our study suggest that the increase in IL-17 expression in the gingival tissue of PD patients occurs at the post-translational stage. Likewise, inflammatory mediators involved in the development of periodontitis cause stimulation of IL-17 protein expression, which increases the amount of IL-17 protein in gingival tissue. Periodontitis is a chronic inflammatory condition in which the immune response is disrupted. Multiple immune response pathways are activated, leading to the synthesis of pro-inflammatory mediators and the formation of numerous feedback loops. These results suggest that IL-17 is part of the inflammatory cascade present in periodontitis. Previous studies have indicated that IL-17 increases the production of other cytokines, so it also appears that other pro-inflammatory mediators may affect IL-17 expression [4,5].

Previous studies have shown increased IL-17 expression in the serum, gingival fluid, and saliva of patients with various forms of periodontal disease [17,18,19,20,21,22]. These studies often used previous criteria for classifying periodontal disease, which may be a factor in the results. In our study, patients were classified according to new criteria in accordance with the 2017 periodontal disease classification system [15]. It has been shown that IL-17 expression can vary in different tissues. IL-17 is demonstrated to be part of a complex inflammatory cascade occurring in periodontitis. There is a mutual influence of individual pro-inflammatory mediators, which interact with each other. Elevated expression of IL-17 protein in gingival tissue may indicate the involvement of IL-17 in the inflammatory process in the periodontal tissues.

Although a number of previous studies have confirmed an important role in the development of periodontal diseases, it seems that a full understanding of the involvement of this cytokine and its individual isoforms in the inflammatory cascade present in periodontitis, especially in light of the new classification of periodontal diseases, requires further research.

## 5. Conclusions

The results of our study confirm the involvement of IL-17 in the pathogenesis of periodontitis.

Our results suggest that an increase in IL-17 protein expression in the gingival tissue of patients with periodontitis occurs at the post-translational stage.

## Figures and Tables

**Table 1:** Correlation between *IL-17A* and *IL-17B* expression and clinical parameters.

	*IL-17A*		*IL-17B*	
Clinical Parameters	Rs	*p*	Rs	*p*
Age [years]	−0.0221	0.94	0.1850	0.52
NRT	−0.0598	0.83	0.0066	0.98
API	0.6099	0.02	0.3300	0.24
BoP	0.0818	0.78	0.0770	0.79
PPD	−0.2539	0.38	−0.0681	0.81
CAL	0.1392	0.63	0.5061	0.06

Rs—Spearman rank correlation coefficient; NRT—Number of remaining teeth; API—approximal plaque index; BoP—bleeding on probing; PPD—probing pocket depth; CAL—clinical attachment level.

## Data Availability

Not applicable.

## References

[1] Kinane D.F., Peterson M., Stathopoulou P.G. (2006). Environmental and other modifying factors of the periodontal diseases. Periodontology 2000.

[2] Di Benedetto A., Gigante I., Colucci S., Grano M. (2013). Periodontal disease: Linking the primary inflammation to bone loss. Clin. Dev. Immunol..

[3] Sandros J., Karlsson C., Lappin D.F., Madianos P.N., Kinane D.F., Papapanou P.N. (2000). Cytokine responses of oral epithelial cells to Porphyromonas gingivalis infection. J. Dent. Res..

[4] Cheng W.C., Hughes F.J., Taams L.S. (2014). The presence, function and regulation of IL-17 and Th17 cells in periodontitis. J. Clin. Periodontol..

[5] Cheng W.C., van Asten S.D., Burns L.A., Evans H.G., Walter G.J., Hashim A., Hughes F.J., Taams L.S. (2016). Periodontitis-associated pathogens P. gingivalis and A. actinomycetemcomitans activate human CD14(+) monocytes leading to enhanced Th17/IL-17 responses. Eur. J. Immunol..

[6] Mitani A., Niedbala W., Fujimura T., Mogi M., Miyamae S., Higuchi N., Abe A., Hishikawa T., Mizutani M., Ishihara Y. (2015). Increased expression of interleukin (IL)-35 and IL-17, but not IL-27, in gingival tissues with chronic periodontitis. J. Periodontol..

[7] Gaffen S.L. (2009). Structure and signalling in the IL-17 receptor family. Nat. Rev. Immunol..

[8] Gu C., Wu L., Li X. (2013). IL-17 family: Cytokines, receptors and signaling. Cytokine.

[9] Miossec P., Kolls J.K. (2012). Targeting IL-17 and TH17 cells in chronic inflammation. Nat. Rev. Drug. Discov..

[10] Zenobia C., Hajishengallis G. (2015). Basic biology and role of interleukin-17 in immunity and inflammation. Periodontology 2000.

[11] Iyoda M., Shibata T., Kawaguchi M., Hizawa N., Yamaoka T., Kokubu F., Akizawa T. (2010). IL-17A and IL-17F stimulate chemokines via MAPK pathways (ERK1/2 and p38 but not JNK) in mouse cultured mesangial cells: Synergy with TNF-alpha and IL-1beta. Am. J. Physiol. Renal. Physiol..

[12] Koenders M.I., Marijnissen R.J., Devesa I., Lubberts E., Joosten L.A., Roth J., van Lent P.L., van de Loo F.A., van den Berg W.B. (2011). Tumor necrosis factor-interleukin-17 interplay induces S100A8, interleukin-1β, and matrix metalloproteinases, and drives irreversible cartilage destruction in murine arthritis: Rationale for combination treatment during arthritis. Arthritis Rheum..

[13] Behfarnia P., Birang R., Andalib A.R., Asadi S. (2010). Comparative Evaluation of IFNγ, IL4 and IL17 Cytokines in Healthy Gingiva and Moderate to Advanced Chronic Periodontitis. J. Dent. Res..

[14] Duarte P.M., Miranda T.S., Lima J.A., Dias Gonçalves T.E., Santos V.R., Bastos M.F., Ribeiro F.V. (2012). Expression of immune-inflammatory markers in sites of chronic periodontitis in patients with type 2 diabetes. J. Periodontol..

[15] Dietrich T., Ower P., Tank M., West N.X., Walter C., Needleman I., Hughes F.J., Wadia R., Milward M.R., Hodge P.J. (2019). Periodontal diagnosis in the context of the 2017 classification system of periodontal diseases and conditions—Implementation in clinical practice. Br. Dent. J..

[16] Papapanou P.N., Sanz M., Buduneli N., Dietrich T., Feres M., Fine D.H., Flemmig T.F., Garcia R., Giannobile W.V., Graziani F. (2018). Periodontitis: Consensus report of workgroup 2 of the 2017 World Workshop on the Classification of Periodontal and Peri-Implant Diseases and Conditions. J. Periodontol..

[17] Ozçaka O., Nalbantsoy A., Buduneli N. (2011). Interleukin-17 and interleukin-18 levels in saliva and plasma of patients with chronic periodontitis. J. Periodont. Res..

[18] Shaker O.G., Ghallab N.A. (2012). IL-17 and IL-11 GCF levels in aggressive and chronic periodontitis patients: Relation to PCR bacterial detection. Mediat. Inflamm..

[19] Vernal R., Dutzan N., Chaparro A., Puente J., Antonieta Valenzuela M., Gamonal J. (2005). Levels of interleukin-17 in gingival crevicular fluid and in supernatants of cellular cultures of gingival tissue from patients with chronic periodontitis. J. Clin. Periodontol..

[20] Awang R.A., Lappin D.F., MacPherson A., Riggio M., Robertson D., Hodge P., Ramage G., Culshaw S., Preshaw P.M., Taylor J. (2014). Clinical associations between IL-17 family cytokines and periodontitis and potential differential roles for IL-17A and IL-17E in periodontal immunity. Inflamm. Res..

[21] Liukkonen J., Gürsoy U.K., Pussinen P.J., Suominen A.L., Könönen E. (2016). Salivary Concentrations of Interleukin (IL)-1β, IL-17A, and IL-23 Vary in Relation to Periodontal Status. J. Periodontol..

[22] Schenkein H.A., Koertge T.E., Brooks C.N., Sabatini R., Purkall D.E., Tew J.G. (2010). IL-17 in sera from patients with aggressive periodontitis. J. Dent. Res..

[23] Qi Y., Feng W., Song A., Song H., Yan S., Sun Q., Yang P. (2013). Role of serum IL-23/IL-17 axis in the relationship between periodontitis and coronary heart disease. Int. J. Periodontics Restor. Dent..

[24] Cifcibasi E., Koyuncuoglu C., Ciblak M., Badur S., Kasali K., Firatli E., Cintan S. (2015). Evaluation of Local and Systemic Levels of Interleukin-17, Interleukin-23, and Myeloperoxidase in Response to Periodontal Therapy in Patients with Generalized Aggressive Periodontitis. Inflammation.

[25] Duarte P.M., da Rocha M., Sampaio E., Mestnik M.J., Feres M., Figueiredo L.C., Bastos M.F., Faveri M. (2010). Serum levels of cytokines in subjects with generalized chronic and aggressive periodontitis before and after non-surgical periodontal therapy: A pilot study. J. Periodontol..

[26] Adibrad M., Deyhimi P., Ganjalikhani Hakemi M., Behfarnia P., Shahabuei M., Rafiee L. (2012). Signs of the presence of Th17 cells in chronic periodontal disease. J. Periodontal. Res..

